# Supplementary material for: Neuropathology of the Basal Ganglia in SNCA Transgenic Rat Model of Parkinson’s Disease: Involvement of Parvalbuminergic Interneurons and Glial-Derived Neurotropic Factor
Source: Int J Mol Sci. 2022 Sep 4;23(17):10126. doi: 10.3390/ijms231710126 (PMC9456397; doi:10.3390/ijms231710126)
Supplement: Supplementary file 1 [file ijms-23-10126-s001.zip › ijms-1905385-supplementary.pdf]

Supplementary figure

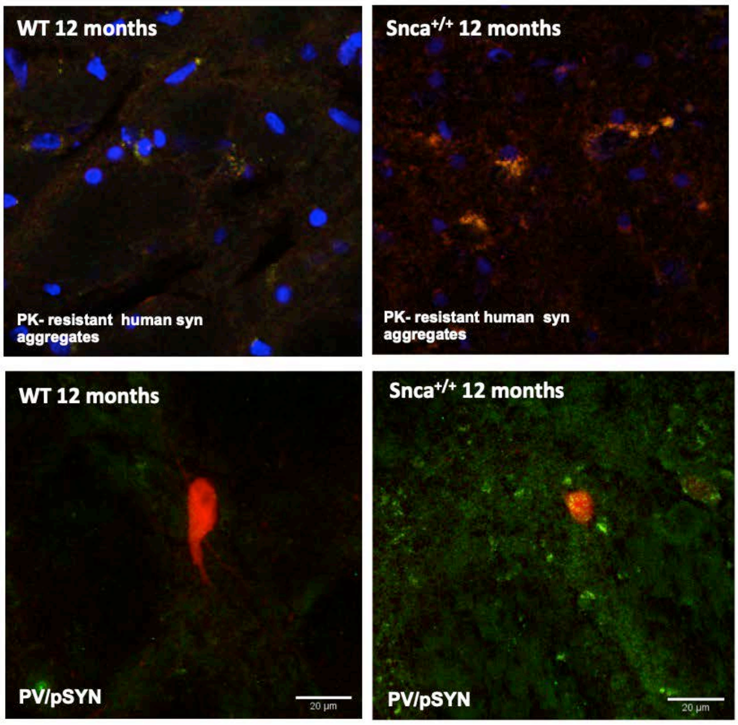

Supplementary Figure S1. Confocal microscope immunofluorescence images show the presence of PK-resistant human syn aggregates only in the 12 months old Snca+/+rats. The 12 months old Snca+/+ also have the phosphorylated form of human Syn in PV+GABAergic Interneurons.
